# Perceptual Decision-Making in Children: Age-Related Differences and EEG Correlates

**DOI:** 10.1007/s42113-020-00087-7

**Published:** 2020-06-19

**Authors:** Catherine Manning, Eric-Jan Wagenmakers, Anthony M. Norcia, Gaia Scerif, Udo Boehm

**Affiliations:** 1 Department of Experimental Psychology, University of Oxford, Oxford, UK; 2 University of Amsterdam, Amsterdam, The Netherlands; 3 Department of Psychology, Stanford University, Stanford, USA

**Keywords:** Diffusion model, Decision-making, Perceptual development, Motion processing, Electroencephalography

## Abstract

Children make faster and more accurate decisions about perceptual information as they get older, but it is unclear how different aspects of the decision-making process change with age. Here, we used hierarchical Bayesian diffusion models to decompose performance in a perceptual task into separate processing components, testing age-related differences in model parameters and links to neural data. We collected behavioural and EEG data from 96 6- to 12-year-old children and 20 adults completing a motion discrimination task. We used a component decomposition technique to identify two response-locked EEG components with ramping activity preceding the response in children and adults: one with activity that was maximal over centro-parietal electrodes and one that was maximal over occipital electrodes. Younger children had lower drift rates (reduced sensitivity), wider boundary separation (increased response caution) and longer non-decision times than older children and adults. Yet, model comparisons suggested that the best model of children’s data included age effects only on drift rate and boundary separation (not non-decision time). Next, we extracted the slope of ramping activity in our EEG components and covaried these with drift rate. The slopes of both EEG components related positively to drift rate, but the best model with EEG covariates included only the centro-parietal component. By decomposing performance into distinct components and relating them to neural markers, diffusion models have the potential to identify the reasons why children with developmental conditions perform differently to typically developing children and to uncover processing differences inapparent in the response time and accuracy data alone.

**Electronic supplementary material:**

The online version of this article (10.1007/s42113-020-00087-7) contains supplementary material, which is available to authorized users.

## Introduction

Children make better decisions about sensory information as they get older. They become able to detect weaker stimuli and make more subtle discriminations between stimuli across a range of tasks. For example, in the visual domain, sensitivity increases during childhood for spatial contrast (Bradley and Freeman 1982; Ellemberg et al. 1999), global motion (Gunn et al. 2002; Hayward et al. 2011; Hadad et al. 2011; Manning et al. 2014), biological motion (Hadad et al. 2011), speed (Manning et al. 2012) and face information (Bruce et al. 2000; Mondloch et al. 2003). Studies of perceptual development typically focus on the accuracy of responses, by defining sensitivity as the stimulus level or stimulus contrast required to sustain performance at a certain level of correct responding (e.g. an 80% correct threshold). Meanwhile, the time taken to respond is not taken into account. This approach fails to make use of all of the available data and means that results could be contaminated by age-related differences in speed-accuracy tradeoffs (Ho et al. 2012; Wickelgren 1977).

**Fig. 1:**
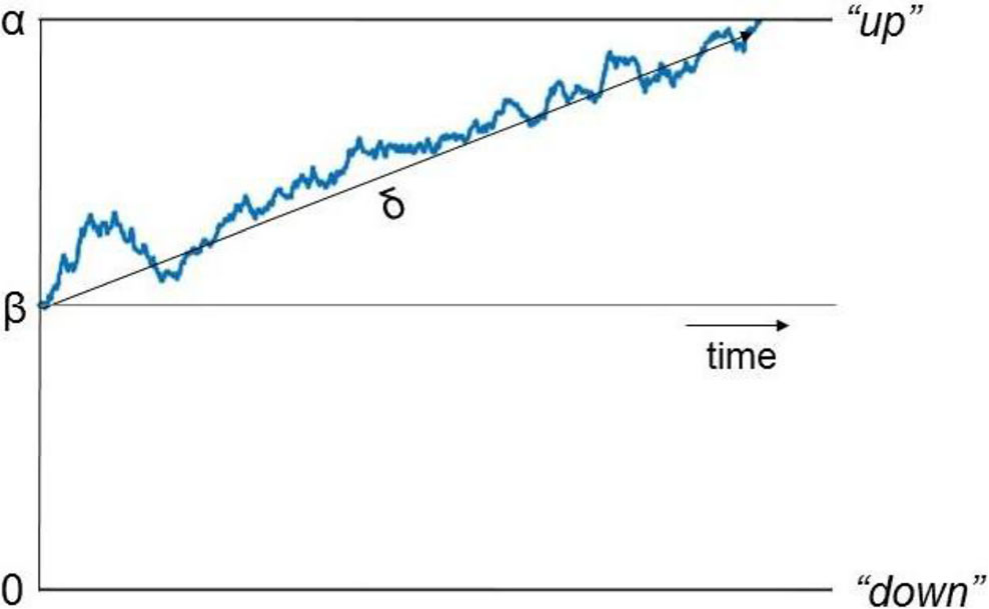
Representation of the decision-making process in the diffusion model. Noisy diffusion process originating from a starting point and approaching one of two decision bounds corresponding to ‘up’ and ‘down’ motion. The drift rate (*δ*) reflects the rate of evidence accumulation towards the decision bounds. The boundary separation (*α*) represents the distance between the two decision bounds, with greater separation suggesting increased response caution. Starting point (*β*) reflects bias towards one of the two decision bounds (here, *β* is 0.5; i.e. the starting point is equidistant between the two bounds)

Here, we analysed both accuracy and response time data using a simplified 4-parameter version of the diffusion model (Ratcliff and Rouder 1998; Ratcliff and McKoon 2008; Stone 1960), which decomposes performance into separate processing components. According to this model, the time taken to respond to sensory information consists of non-decision processes (including sensory encoding and action generation) and a decision process. The time taken for non-decision processes is combined into a single estimate of non-decision time (*τ*). The decision process is modelled as a stochastic process in which noisy sensory information is accumulated towards one of two decision bounds corresponding to different responses (Fig. 1). In a direction discrimination task, the bounds could correspond to ‘up’ and ‘down’ responses, for example. The drift rate reflects the within-trial average rate of evidence accumulation (*δ*) (Fig. 1), which is determined by the quality of sensory evidence (e.g. higher drift rates will be obtained if there is a higher signal-to-noise ratio in a stimulus) and the observer’s sensitivity to a given stimulus. The distance between the decision bounds reflects the criterion for responding (i.e. how much evidence is required to trigger a response). Wider decision bounds indicate increased response caution, whereas narrow decision bounds indicate increased urgency. If the starting point of the decisional process (*β*) (Fig. 1) is closer to one decision bound than the other, this indicates response bias. Individuals can differ in their drift rates, decision bounds, biases and non-decision time (as well as in the variability of these parameters, when fitting the full model). Therefore, diffusion modelling allows better identification of the sources of difference between individuals or groups than when comparing accuracy or response times alone, while also accounting for potential speed-accuracy tradeoffs (White et al. 2010).

When applied to cross-sectional studies of visual development, the diffusion model allows the nature of age-related differences to be investigated more carefully. Specifically, it is possible to address whether young children show reduced sensitivity to visual information due to a slower rate of accumulation of sensory evidence, while controlling for differences in criterion setting (decision bounds) and non-decisional processes (Wagenmakers et al. 2007; White et al. 2010). An additional benefit of this modelling approach is that the resulting processing components are likely to correspond more directly to neural mechanisms than accuracy or response time measures (White et al. 2016; Forstmann et al. 2016), thus potentially facilitating links between neural and behavioural data on visual development.

Diffusion models have not yet been routinely applied to understanding perceptual development in children—perhaps because these models typically require many trials. Ratcliff et al. (2012) successfully fitted diffusion models to data obtained in numerosity discrimination and lexical decision tasks in children and adolescents aged 8 to 16 years, across 1200 trials. Young people were less accurate and had longer response times than adult participants. Diffusion modelling showed that these behavioural changes were accounted for by age-related differences in multiple model parameters. Young people had lower drift rates than adults, meaning that they extracted information from the stimulus more slowly. They also had wider response bounds and increased non-decision time than adults, as well as increased variability in non-decision time. This pattern of results was qualitatively different from one related to ageing: while older adults also showed wider decision bounds and longer non-decision times compared to younger adults, they did not differ in their drift rates (Ratcliff et al. 2004).

Recent methodological advances mean that diffusion models can now be fit using fewer trials. Hierarchical approaches make efficient use of the statistical structure available across participants and conditions, so that information at the group level informs estimates for individual participants (Vandekerckhove et al. 2011; Wiecki et al. 2013). When compared with models which fit parameters for each individual separately, hierarchical diffusion models perform well, particularly when trial numbers are low (Ratcliff and Childers 2015). Therefore, hierarchical models have clear advantages for assessing young children, for whom extensive trial numbers cannot be obtained. Indeed, they have been used to assess the performance of children as young as 3 years old in language processing tasks, with as few as 30 trials (Nordmeyer et al. 2016; Schneider and Frank 2016). These findings suggest that young children have shallower drift rates, higher boundary separation and longer non-decision times than adult participants, in line with the findings of Ratcliff et al. (2012).

Within hierarchical models, it is also possible to investigate relationships between model parameters and neurophysiological measures from techniques such as electroencephalography (EEG), functional magnetic resonance imaging (fMRI) and eye tracking (e.g. Cassey et al. 2014, 2016; Turner et al. 2013, 2015, 2017). The inclusion of such measures in the model helps to inform the neural correlates of parameters and bridge brain and behavioural data (Ratcliff and McKoon 2008; Turner et al. 2016). While there are a range of ways in which neural and behavioural data can be combined (see Turner et al. 2016 for review), in this study, we used a regression approach in which the slope of the model parameter (e.g. drift rate) is weighted by the value of the neurophysiological measure (Boehm et al. 2017). Establishing such links is important to ensure that the parameters of the diffusion model are psychologically meaningful rather than merely abstract concepts. EEG is a particularly useful tool in this context for two reasons. First, it has a fine-grained temporal resolution that allows distinct components of the decision-making process to be disentangled (Kelly and O’Connell 2015). Second, EEG provides a global view of brain function which complements current understandings of decision-related activity being represented broadly across the brain (Dmochowski and Norcia 2015; Katz et al. 2016; Mulder et al. 2014).

The current study aims to increase our understanding of perceptual decision-making across childhood, using hierarchical diffusion models with both behavioural and electrophysiological data. To our knowledge, this is the first study to combine behavioural and electrophysiological measures in diffusion models in children, and will enable new insights into the mechanisms of perceptual development. A motion coherence task was used, for three reasons. First, this task is commonly used in decision-making studies, by virtue of the ease in which the strength of dynamic sensory evidence can be manipulated by varying coherence and the relatively long response times elicited (Gold and Shadlen 2007; Kelly and O’Connell 2015). Second, these tasks have been previously used with children and have been shown to be sensitive to developmental change throughout childhood (Gunn et al. 2002; Hayward et al. 2011; Hadad et al. 2011; Manning et al. 2014). Third, much research has been conducted into the electrophysiological correlates of diffusion model parameters using this task (e.g. Dmochowski and Norcia 2015; Kelly and O’Connell 2013).

Initially, the motion coherence task was used to investigate single-cell activity related to the decision process in monkeys. The ramping up of activity in cells in lateral intraparietal cortex (LIP) tracks the accumulation of sensory evidence, with steeper ramping activity and higher levels of activity at the end of the stimulus period being associated with higher levels of motion coherence (Shadlen and Newsome 1996, 2001). Cells in the medial temporal (MT) cortex are also activated during the motion coherence task (Shadlen and Newsome 2001), but the firing of these cells most likely reflects momentary evidence, which is then integrated in LIP to reflect the drift rate (Hanks et al. 2006). Evidence accumulation activity has also been demonstrated in another parietal area, the medial intraparietal (MIP) area, when monkeys are required to make their response by reaching (de Lafuente et al. 2015).

Electrophysiological correlates of decision-making processes have also been investigated in human observers during motion coherence tasks, using EEG. Kelly and O’Connell (2013) reported that the centro-parietal positivity (CPP) reflected evidence accumulation, with higher rates of build-up when the motion coherence was high. Using a subtly different motion processing task in which the strength of sensory evidence was manipulated by increasing the amount of variability in dot directions, Dmochowski and Norcia (2015) identified a CPP-like component using reliable components analysis (Dmochowski et al. 2012, 2014). Like the CPP, this component was maximal over the medial parietal cortex and evidence-dependent, with steeper ramping activity in trials with more sensory evidence (i.e. easier trials) and with ramping activity correlating with response time. The authors identified two further components with ramping activity which were relevant to the decision-making process. These adult studies suggest candidate neural correlates of diffusion model parameters for our study with children.

### Research Aims

The aim of the current study was to characterise age-related changes in diffusion model parameters. We expected drift rates to increase with age, in line with previous reports of age-related increases in sensitivity to visual motion across childhood measured using psychophysical thresholds. Previous studies applying diffusion models to children’s performance in numerosity discrimination, lexical decision and linguistic comprehension tasks have shown that children have higher boundary separation and longer non-decision times than adults, in conjunction with lower drift rates (Nordmeyer et al. 2016; Ratcliff et al. 2012; Schneider and Frank 2016). Here, we assess whether these findings generalise to our visual motion processing task. We also investigate age-related differences in neural correlates using high-density EEG. Based on adult studies, we expected to see ramping activity in centro-parietal electrodes, reflecting the rate of evidence accumulation. We aimed to directly assess this relationship by linking model parameters to electrophysiological measures using a Bayesian regression approach (Boehm et al. 2017).

## Methods

### Participants

We analysed the data from 96 children (44 females) aged 6 to 12 years (*M* = 9.25 years, SD = 2.05 years) and 20 adults (9 females) aged between 19 and 35 years (*M* = 27.65 years, SD = 5.43 years), with no history of developmental conditions and normal or corrected-to-normal vision (assessed with a Snellen chart). Two additional child participants were excluded as they gave reverse responses to motion direction, responding substantially *below* chance, resulting in negative drift rates. Four further child participants were removed as only 3.7%, 39.0%, 53.6% and 55.9% of their data survived data cleaning using the exponential weighted moving average method (see below), suggesting that they were making a high proportion of fast guesses. Our analysis used the same dataset that has been analysed previously in a study focusing on children’s electrophysiological responses locked to the onset of coherent motion (Manning et al. 2019). The data are available in a figshare collection (Manning et al. 2020).

### Apparatus

The task was presented on a Dell Precision M3800 laptop (2048 × 152 pixels, 60 Hz) using the Psychophysics Toolbox for MATLAB (Brainard 1997; Kleiner et al. 2007; Pelli 1997). EEG signals were collected with 128-channel HydroCel Geodesic Sensor Nets connected to Net Amps 300 (Electrical Geodesics, Inc., OR, USA), using NetStation 4.5 software. A photodiode attached to the monitor independently verified stimulus presentation timing. Participants responded using a Cedrus RB-540 response box (Cedrus, CA, USA).

### Stimuli

**Fig. 2:**
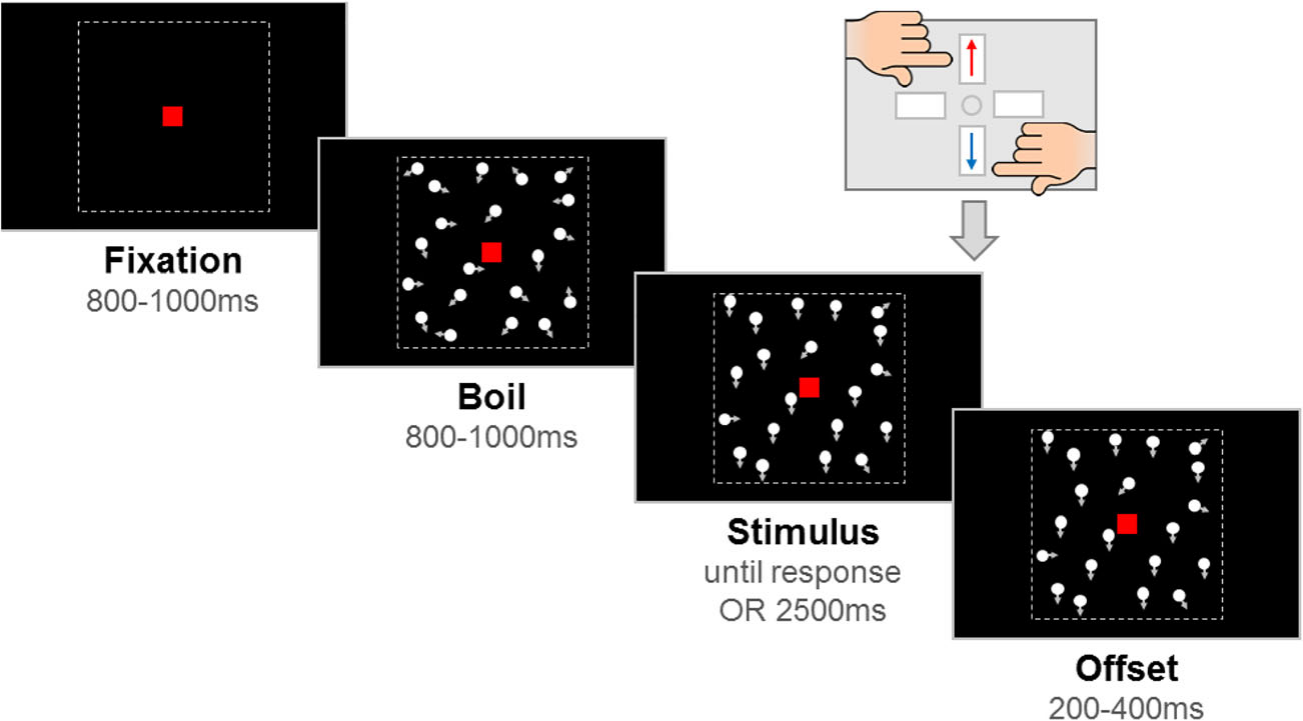
Schematic representation of trial procedure. A *fixation* period was followed by a *boil* period consisting of random, incoherent motion. The *stimulus* appeared containing coherent motion, and the participant was required to report the direction (up, down) using a response box. The stimulus period lasted until a response was made, or until 2500 ms elapsed. Coherent motion remained on the screen for an *offset* period. The durations of the fixation, boil and offset periods were jittered within a fixed range to minimise expectancy effects. N.b. arrows (indicating movement) and dotted lines (marking the square aperture) are presented for illustrative purposes, only (figure reproduced from Manning et al. 2019)

We presented random-dot stimuli comprised of 100 white dots (diameter 0.19°) moving within a square aperture (10° × 10°) against a black screen. The dots moved at a speed of 6°/s and had a limited lifetime of 200 ms. A red, square-shaped fixation point (0.24° × 0.24°) was presented throughout the trial. Each trial consisted of a fixation period, a boil period, a stimulus period and an offset period (see Fig. 2). The fixation period had a randomly selected duration between 800 and 1000 ms. The boil period presented the stimulus dots moving in random, incoherent directions, for a randomly selected duration between 800 and 1000 ms. The boil period ensured that pattern-evoked and motion-onset-evoked potentials did not overlap temporally with the onset of evidence (directional, coherent motion) required for the perceptual decision. In the stimulus period, we introduced coherent motion (either upward or downward) in a proportion of dots, while the remainder of the dots continued to move in random directions. The stimulus period was presented until a response was made, or until 2500 ms had elapsed. Finally, the offset period continued the stimulus presentation for a randomly selected duration between 200 and 400 ms, which served to dissociate response-locked EEG activity from that associated with stimulus offset.

### Experimental Task Procedure

The task was presented in the context of a child-friendly game set in ‘Insectland’. Using animations, it was explained to participants that the fireflies were escaping from their viewing boxes, and that the zookeeper needed their help to know whether the fireflies were escaping out through the top or the bottom of the boxes, so that he could fix them and ‘save’ the fireflies. Participants were told that there were 10 different viewing boxes, corresponding to 10 different ‘levels’ of the game. Level 1 was a practice phase to familiarise participants with the task demands. Initially, four demonstration trials were presented with no boil phase and an unlimited stimulus phase, which the experimenter used to explain the task to participants. Participants were told to report the direction using the ‘up’ and ‘down’ arrow keys on the response box. Half of the participants were instructed to use their left hand on the ‘up’ key and their right hand on the ‘down’ key, and vice versa for the other participants. The first two demonstration trials moved with 100% coherence, and the last two moved with 75% and 50% coherence, respectively, to explain to participants that sometimes not all of the fireflies would escape, so in this case, they would have to say where most of the fireflies were going.

Following the demonstration trials, there were up to 20 criterion trials with a coherence of 95%. These trials introduced the boil phase. It was explained to participants that the fireflies would be going ‘all over the place’ at first, so that they must wait for the fireflies to escape before responding. A time limit was enforced at this point, with visual feedback presented on the screen if participants did not respond within 2500 ms (‘Timeout! Try to be quicker next time!’, presented in red text). When participants met a criterion of four consecutive correct responses, no more criterion trials were presented. Next, there were eight practice trials of increasing difficulty (80%, 70%, 60%, 50%, 40%, 30%, 20%, 10%). Participants were reassured that it was fine if they got some of these wrong and/or if they had to guess. Visual feedback was presented after each trial throughout level 1 (‘That was correct!’, presented in green text, or ‘It was the other way that time’ or ‘Timeout! Try to be quicker next time!’, presented in red text). For 10 children, level 1 was repeated as they did not meet the criterion of four consecutive correct responses on the first attempt.

Levels 2–10 each contained 36 trials, with 4 repetitions of each of four coherence levels (10%, 30%, 50%, 75%), for each coherent direction (upward, downward), and an additional 4 catch trials presenting 100% coherent motion. Thus, the experimental phase consisted of 324 trials in total. The order of trials within each block was randomised. No trial-by-trial feedback was presented during the experimental phase, apart from a ‘timeout’ message (as before) if no response was provided within 2500 ms of the stimulus period. At the end of each level, participants were presented with their ‘points’ for the preceding block of trials. Participants were told that they would get points for both going fast and getting as many right as possible. The points reflected an efficiency score, computed by 1 / median response time × accuracy, rounded to the nearest integer. In the event that participants obtained an efficiency score under 10, a score of 10 points was given to avoid demotivation. Trials were presented automatically, although the experimenter had controls to pause and resume trial presentation (see below). The experimental code can be found at https://osf.io/fkjt6/.

### General Procedure

The procedure was approved by the Central University Research Ethics Committee at the University of Oxford. Adult participants and parents of child participants provided written informed consent, and children provided verbal or written assent. Participants initially completed a Snellen acuity test to confirm normal or corrected-to-normal vision. The sensor net was then placed on the participant, and the experimenters ensured that electrode impedances were below 50 kΩ. EEG data were acquired at a sampling rate of 500 Hz with a vertex reference electrode. During the experimental task, participants sat 80 cm away from the computer screen in a darkened room. Children were closely monitored by an experimenter sitting beside them. The experimenter provided general encouragement and task reminders, pausing before the start of a trial where necessary (e.g. to remind the child to keep still). Children had short breaks at the end of each block (or ‘level’) of the experimental task, and a longer break halfway through the experimental task (i.e. at the end of ‘level 5’), at which point the EEG recording was paused and electrode impedances were re-assessed. Note that for two children, impedance checks were completed at the end of level 4 or level 6, as appropriate. Children marked their progress through the levels using a stamper on a record card. The whole session took no longer than 1.5 h. Adult participants were paid £15, and children were given a £5 gift voucher to thank them for their time.

### Data Screening

First, we removed the catch trials (with 100% coherence), because (a) they were fewer in number than the experimental trials and (b) some participants performed less accurately in these trials than the trials in the 75% coherence condition, suggesting that they were using a different strategy in the catch trials. We also removed trials in which no response was made within 2500 ms. Preliminary analysis revealed that data from the hardest (10% coherence) condition were not well fit by a standard diffusion model (see posterior predictive fits in Supplementary Figures S1 and S2), which could be due to a series of explanations, such as deadline effects, temporal uncertainty about the stimulus and lack of confidence. Therefore, we decided to exclude the data from this condition and to continue the analysis with three conditions (30%, 50% and 75% coherence).

We used the exponential weighted moving average method to filter out fast guesses (Vandekerckhove and Tuerlinckx 2007; Nunez et al. 2017). This method involved sorting each participant’s trials (across all 3 conditions) by response time and then determining the shortest response time at which the participant was responding at above-chance accuracy (i.e. when the response times deviated from a ‘control’ process governed by chance performance). This was achieved by computing the EWMA statistic (*c*) for each sorted trial (*s*)

1 $$ {c}_s=\lambda {x}_s+\left(1-\lambda \right){c}_{s-1} $$

where *x*_*s*_ was the response accuracy of trial *s*, and *λ* was a weight parameter determining how many of the preceding trials were used, which we set to 0.1. For each sorted trial, the upper control limit (UCL) of the process was computed

2 $$ {\mathrm{UCL}}_s={c}_0+L{\sigma}_0\sqrt{\frac{\lambda }{2-\lambda}\left[1-{\left(1-\lambda \right)}^{2s}\right]} $$

where *c*_0_ and *σ*_0_ were the mean and standard deviation of the control process, respectively. We set *c*_0_ to 0.5 (reflecting the 50% accuracy expected in the control state) and *σ*_0_ was 0.5 (following the properties of a Bernoulli distribution). *L* was the width of the control limits in standard deviations, which we set to 1.5. We then compared UCL_*s*_ with *c*_*s*_. When *c*_*s*_ was greater than UCL_*s*_, it suggested that the probability of giving a correct response was significantly above chance (> 0.5) for trial *s*. Trials with response times shorter than trial *s* were excluded from analysis. Participants with < 60% of data retained were removed from the dataset (4 children). Of the retained participants, an average of 89.7% of trials (range 71.0–95.4%) was retained for the child participants and 94.1% of trials (range 88.4–95.4%) were retained for the adult participants.

### Diffusion Modelling

We coded ‘up’ responses as hitting the upper boundary and ‘down’ responses as hitting the lower boundary (see Fig. 1), with the match between the stimulus direction and response reflecting response accuracy. Choice and response time data were combined into a bivariate vector *y*. According to the 4-parameter diffusion model, the data *y* are generated by a process with the following parameters: drift rate (*δ*), starting point bias (*β*), boundary separation (*α*) and non-decision time (*τ*). The diffusion coefficient was set to 1 (Wabersich and Vandekerckhove 2014). Note that we used this 4-parameter version of the diffusion model rather than the full diffusion model as between-trial variability parameters are difficult to identify, particularly with few trials (Boehm et al. 2018; Lerche and Voss 2017; Ratcliff and Childers 2015). We sampled from the joint posterior distribution using Just Another Gibbs Sampler (JAGS; Plummer 2003) with the Wiener diffusion distribution (Wabersich and Vandekerckhove 2014), interfaced with R using the *R2jags* package (Su and Yajima 2015).

**Fig. 3:**
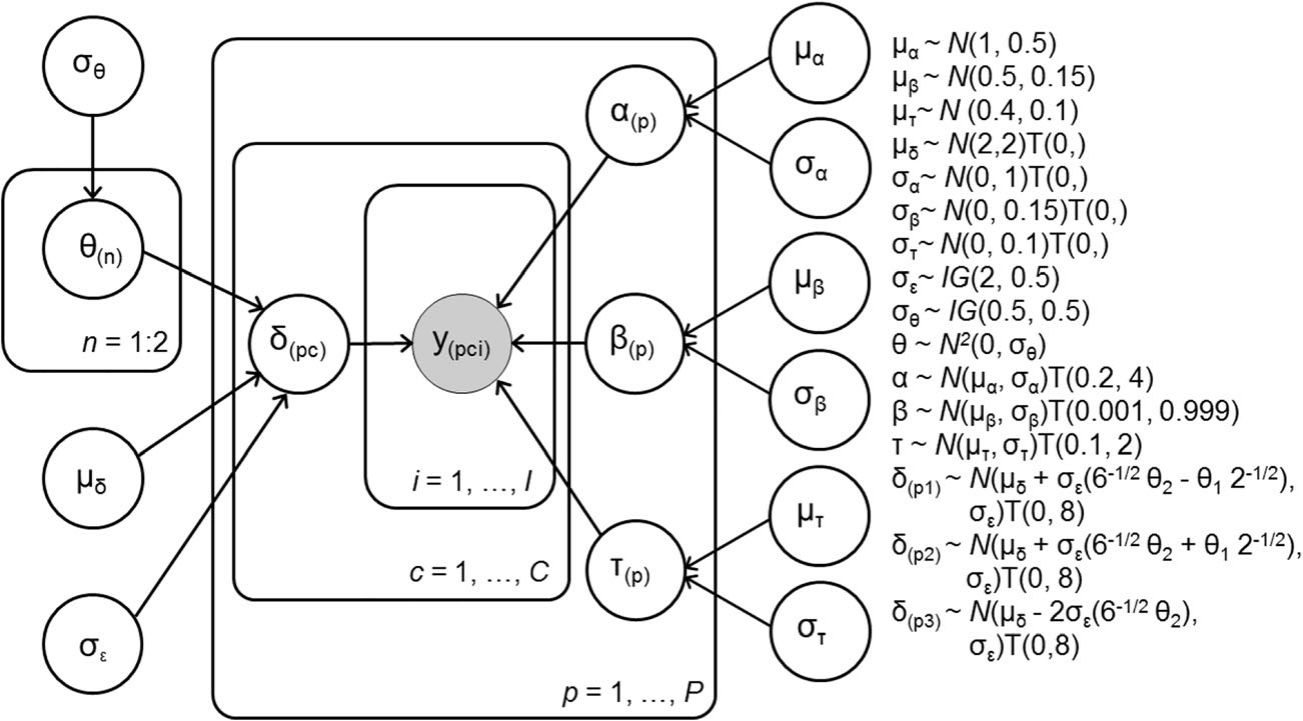
Graphical representation of model 1

We first fit a hierarchical model to the behavioural data for the children and adults separately (model 1). A graphical representation of model 1 is provided in Fig. 3. The data *y*_*pci*_ for each participant (*p*), each condition (*c*) and each trial (*i*) were assumed to be distributed according to the diffusion model’s first passage time distribution

3 $$ {y}_{pc i}\sim \mathrm{Wiener}\left({\alpha}_p,{\beta}_p,{\tau}_p,{\delta}_{pc}\right) $$

where boundary separation (*α*), starting point (*β*) and non-decision time (*τ*) were assumed to vary across participants but assumed to be constant across conditions. As drift rate is known to vary as a function of motion coherence (Kelly and O’Connell 2013; Ratcliff and McKoon 2008), drift rate (*δ*) was allowed to vary both across participants and conditions. We adopted the objective Bayesian regression framework suggested by Liang et al. (2008) (see also Rouder and Morey 2012) and its extension to ANOVA models by Rouder et al. (2012) to account for fixed condition effects and random participant effects. Specifically, we modelled the fixed effect of experimental condition on drift rate by imposing the sum-to-one constraint suggested by Rouder et al. (2012) on the standardised effect sizes (*θ*_(*n*)_) (*n* = 1, 2). That is, the drift rate for participant *p* in condition *c* was drawn from a truncated normal distribution (constraining drift rate to be positive) with a mean determined by the population mean (*μ*_*δ*_) and the standardised effect sizes (*θ*_1_ and *θ*_2_) and standard deviation (*σ*_*ε*_) (see Fig. 3). We modelled the variation between participants as random effects on boundary separation, starting point and non-decision time, where the model parameter for participant *p* was drawn from a truncated normal distribution with mean *μ*_*α*/*β*/*τ*_ and standard deviation *σ*_*α*/*β*/*τ*_. These group-level distributions for the model parameters were truncated to ensure plausibility. The normal distribution on boundary separation (*α*) was truncated between 0.2 and 4, the distribution on starting point bias (*β*) was truncated between 0.001 and 0.999, the distribution on non-decision time (*τ*) was truncated between 0.1 and 2 and the normal distribution on drift rates for each experimental condition (*δ*_*c*_) was truncated between 0 and 8. Priors for the group-level means (*μ*_*α*_, *μ*_*β*_, *μ*_*δ*_, *μ*_*τ*_) and variances (*σ*_*α*_^2^, *σ*_*β*_^2^, *σ*_*τ*_^2^) were informative priors based on Matzke and Wagenmakers’ (2009) survey of parameter values estimated in earlier studies.

We fit further models in which we assessed the relationship between model parameters and age and EEG covariates. Following the Bayesian regression framework for cognitive models suggested by Boehm et al. (2017), we used z-standardised age and EEG covariates and specified mixture of g-priors for the regression weights (Liang et al. 2008; Consonni et al. 2018). In each model, we added a regression term involving a covariate or set of covariates to the fixed effects model for drift rate. Model comparisons were based on the deviance information criterion (DIC) (Spiegelhalter et al. 2002), with preference given to models with lower values.

For each model, we sampled from the posterior distribution using 3 parallel chains each with 54,000 samples and a burn-in period of 4000 samples which were later discarded. We also thinned each chain to save memory space, retaining only every fifth sample, which resulted in 10,000 retained samples per chain (i.e. 30,000 samples in total). Starting values for all parameter values were drawn from uniform distributions that were adjusted to the range of admissible parameter values. The model files and analysis scripts can be found at 10.6084/m9.figshare.c.5006684.

### EEG Data Pre-Processing

The data were pre-processed as described in Manning et al. (2019; for pre-processing scripts, see https://osf.io/fkjt6/). Briefly, the data were epoched into trials, and the data from each trial were median-corrected for DC offsets. We plotted a histogram of the absolute amplitude of each participant’s data across samples and electrodes and identified outliers as those exceeding the 97.5th percentile. We removed electrodes from a half-session (before and after the break when impedances were checked) if they contained ≥ 15% samples exceeding the 97.5th percentile and replaced them with an average of the nearest 6 neighbouring electrodes (or the nearest 4 neighbours for electrodes on the net’s perimeter). We then regressed out horizontal and vertical electrooculogram (EOG) from each electrode. We then replotted histograms of the absolute amplitude for each individual and recalculated the 97.5th percentile, and we removed electrodes for each trial in which ≥ 15% of samples exceeded this cut-off. Next, we removed transients (samples that were four or more standard deviations away from the electrode’s mean) and replaced these with missing values. The data were converted to the average reference and baselined to the average of the last 100 ms of the boil period. In trials where data were removed from 19 or more (≥ 15%) electrodes, we excluded the EEG data completely.

### EEG Analysis

As in Manning et al. (2019), we used a dimensionality reduction technique—reliable components analysis (RCA) (Dmochowski et al. 2012, 2014; Dmochowski and Norcia 2015)—to identify components that maximised spatiotemporal trial-to-trial reliability. These components are sets of electrode weights that can be projected through a forward-model projection to visualise components as scalp topographies (Haufe et al. 2014; Parra et al. 2005). Data projected through these weights can be averaged for each timepoint to provide a time course for the component. Unlike traditional event-related potential analysis, this approach identifies topographic regions of interest using the whole electrode array in a data-driven fashion, while increasing the signal-to-noise ratio as each component represents a weighted average of electrodes. To extract response-locked reliable components, we first re-epoched the trial data to select the 600 ms preceding the response to 200 ms after the response. We then submitted these response-locked data to RCA for the children and adults separately, to get two sets of weights. Initial analyses revealed no lateralised components, so we analysed the data for left- and right-handed responses together.

### Extracting EEG Covariates

**Fig. 4:**
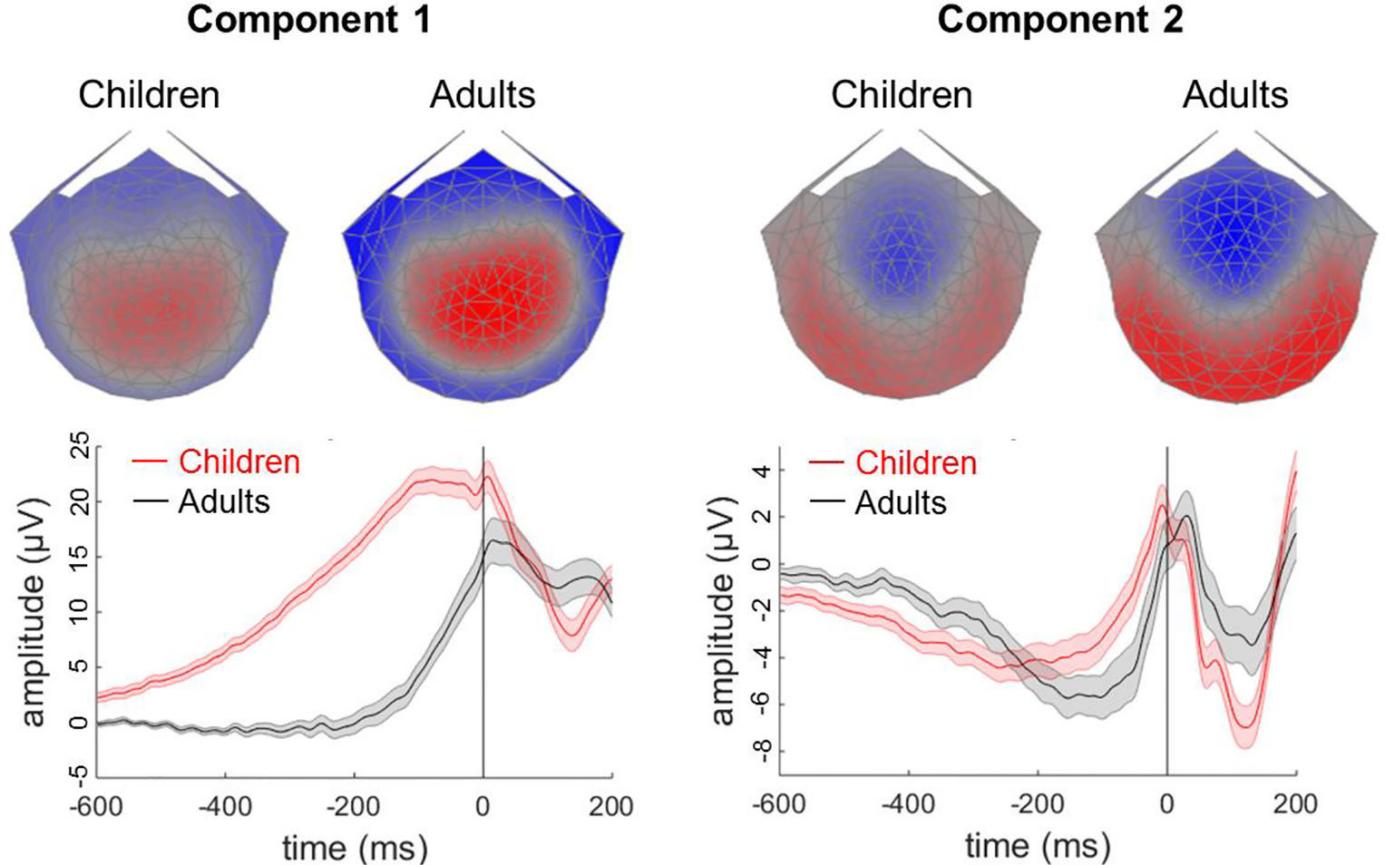
Scalp topographies and component grand average waveforms for response-locked reliable components 1 and 2. Topographic visualisations of the forward-model projections of components 1 and 2 (upper panel) reflecting the weights given to each electrode following RCA for children and adult participants (upper panels). The grand average waveforms (lower panel) show the data multiplied by these spatial weights, for children (red) and adults (black). Shaded error bars represent the standard error of the mean

The first two response-locked components extracted by RCA shared similar topographies for children and adults, with component 1 being maximal over centro-parietal electrodes and component 2 being maximal over occipital electrodes (Fig. 4). These components together explained 76% of the total trial-by-trial reliability in adults (63% for component 1, alone) and 61% of the total trial-by-trial reliability in children (45% for component 1, alone). These components were similar to the response-locked components reported in adult participants by Dmochowski and Norcia (2015), with ramping activity preceding the response in both components. As shown in Fig. 4, this ramping activity was shallower in children than in adults.

The gradient of ramping activity in similar components has been related to drift rates, with steeper slopes reflecting higher drift rates (Dmochowski and Norcia 2015; Kelly and O’Connell 2013). Therefore, we aimed to extract the gradient by fitting a regression line to each participant’s averaged activity for each component, within a given regression window, which could then be entered as a covariate relating to drift rate in the model. As can be seen in the grand average waveform (Fig. 4), the onset of the ramping activity in component 1 was not well-defined and the peak was relatively broad (particularly for children), so we used a multi-stage process to select regression windows appropriate for the child and adult groups. First, we identified the peak from the grand average waveform for children and adults separately using the mid-latency procedure (Picton et al. 2000). For each group, we calculated the 90% peak amplitude and found the latencies corresponding to this 90% amplitude either side of the peak, and then averaged them to find the mid-latency. The mid-latency was used to define the end of the regression window. Next, we found the 30% amplitude of this mid-latency amplitude in the grand average waveform to define the start of the regression window. The resulting regression windows for component 1 were between − 398 and − 52 ms for children and between − 94 and 32 ms for adults.

For component 2, the grand average waveforms were characterised by a negative peak followed by a positive peak close to the response (Fig. 4). We therefore defined the regression window as the period between these two peaks. To select these peaks, we identified the maximum positive amplitudes and maximum negative amplitudes in the grand average waveforms between − 600 and 100 ms (as there were larger peaks from ~ 100 ms after the response). We used the mid-latency procedure for both peaks, averaging across the latencies corresponding to the 90% amplitude of each peak. The mid-latency for the negative peak was taken as the start of the regression window, and the mid-latency for the positive peak was taken as the end of the regression window. The resulting regression windows for component 2 were between − 226 and − 8 ms for children and between − 132 and 30 ms for adults. Linear regression lines were then fit to each participant’s average waveform within the corresponding regression windows for components 1 and 2, and the slope values were entered as covariates in the diffusion model (see Table 1).

**Table 1:** Model comparison statistics

Model	Covariates	DIC (Δ DIC)	
		Children	Adults
Basic model with no covariates			
Model 1	None	13,862.0 (10.9)	− 3641.6 (0.9)
Models assessing age-related effects			
Model 2	*δ*: age *α*: age *τ*: age	13,854.3 (3.2)	
Model 3	*δ*: age	13,863.8 (12.7)	
Model 4	*δ*: age *α*: age	13,851.1 (0)	
Model 5	*δ*: age *τ*: age	13,861.9 (10.8)	
Models assessing EEG correlates			
Model 6	*δ*: EEG1	13,863.1 (12)	− 3635.6 (6.9)
Model 7	*δ*: EEG2	13,855.9 (4.8)	− 3642.5 (0)
Model 8	*δ*: EEG1, EEG2	13,857.3 (6.2)	− 3636.8 (5.7)
Models assessing both age and EEG effects			
Model 9	*δ*: age, EEG1, EEG2	13,875.9 (24.8)	
Model 10	*δ*: age, EEG1, EEG2 *α*: age	13,863.5 (12.4)	
Model 11	*δ*: age, EEG1 *α*: age	13,866.2 (15.1)	
Model 12	*δ*: age, EEG2 *α*: age	13,857.1 (6)	

Values in brackets are the difference in DIC values (Δ DIC) from the model with the lowest DIC value (model 4 for the children and model 7 for the adults). Models including age as a covariate are not presented for adults as no age-related effects were expected in these participants. *δ* = drift rate; *α* = boundary separation; *τ* = non-decision time; EEG1 = slope of EEG reliable component 1; EEG2 = slope of EEG reliable component 2

*DIC* deviance information criterion

## Results

### Age-Related Differences in Accuracy and Response Time

**Fig. 5:**
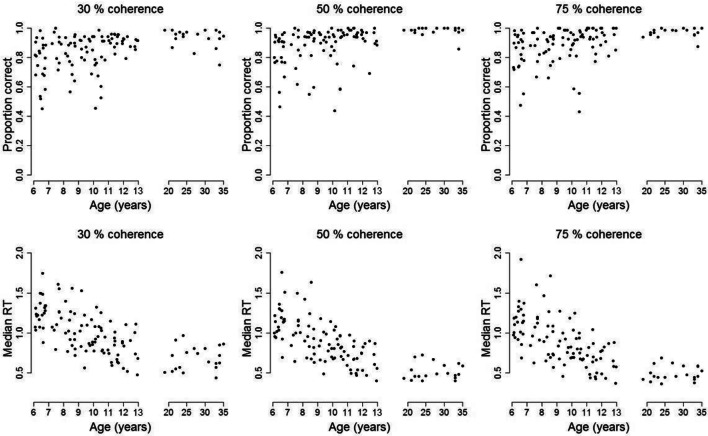
Accuracy and median response times for participants as a function of age

There were age-related differences in accuracy and response time, as shown in Fig. 5, with older children and adults having faster median response times and higher accuracies than younger children. Overall, the mean of median response times in the child group was 0.934 s (95% CI = [0.877, 0.991]) and the mean accuracy was 0.874 (95% CI = [0.869, 0.879]), whereas the adults had a faster mean of median response times (0.618 s, 95% CI = [0.609, 0.627]) and higher accuracy levels (*M* = 0.963, 95% CI = [0.957, 0.968]).

### Differences in Model Parameters Between Children and Adults

**Fig. 6:**
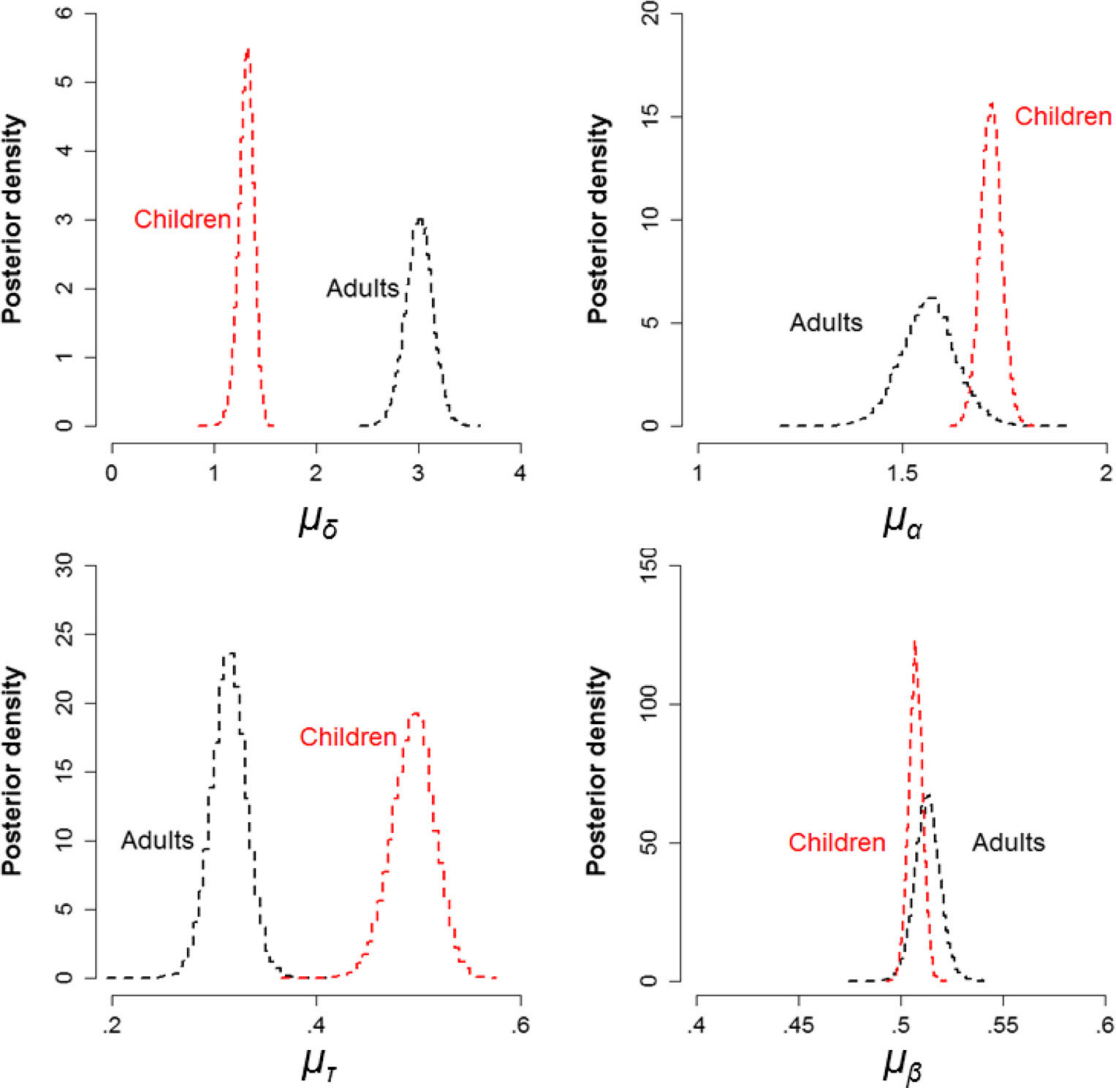
Group mean posteriors for diffusion model parameters (drift rate (*δ*), boundary separation (*α*), non-decision time (*τ*) and starting point (*β*)) for children (red) and adults (black)

**Fig. 7:**
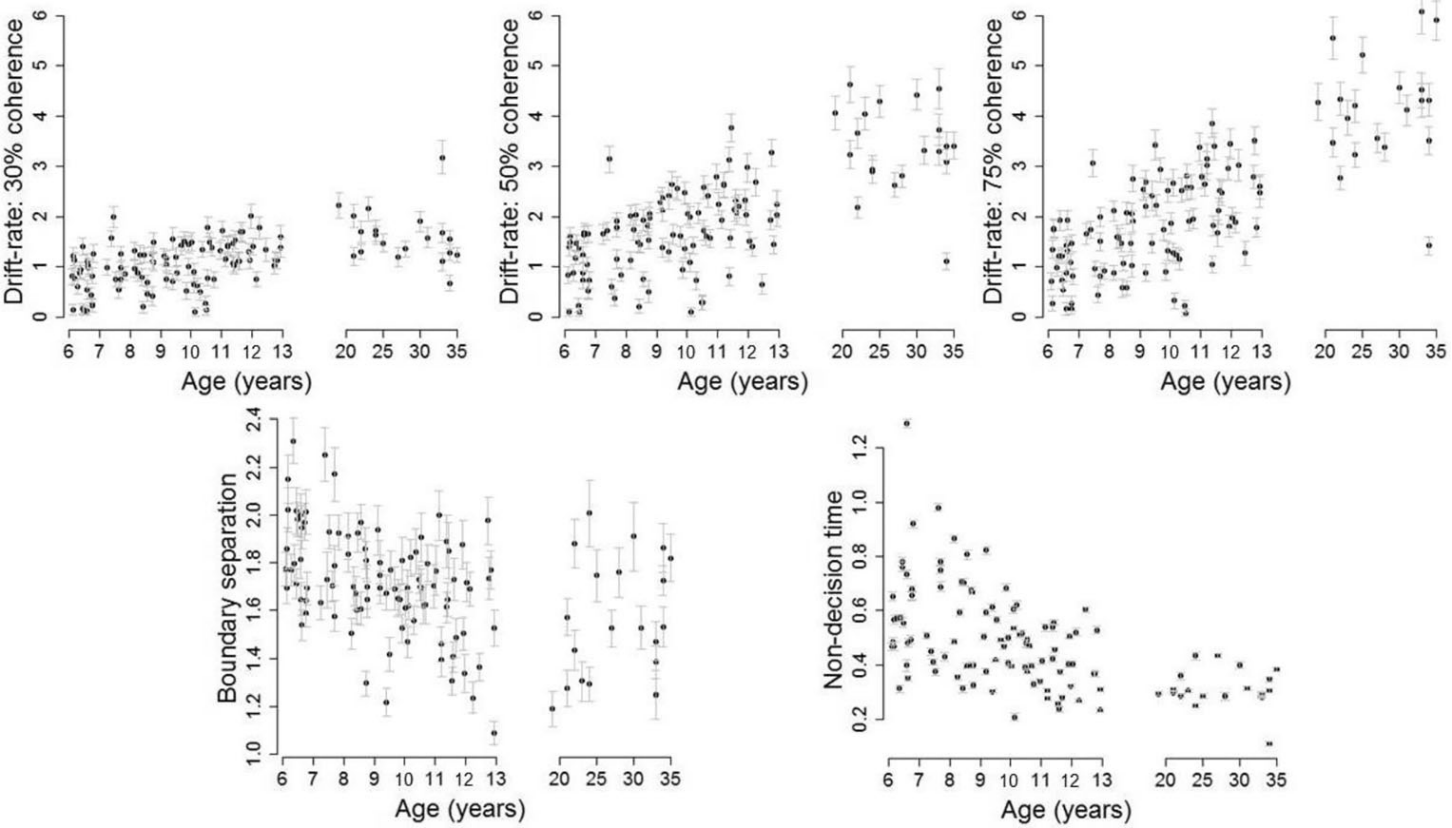
Diffusion model estimates for each participant as a function of age. Mean ± 1 SD of the posterior distribution for each participant’s drift rate, boundary separation and non-decision time parameters

All chains for all models converged well, as indicated by Gelman-Rubin diagnostic values (Gelman and Rubin 1992) close to 1 (range = 0.999–1.028). Posterior predictives for the basic model without covariates (model 1, Fig. 3) are presented in Supplementary Figures S3 and S4, and posterior predictive plots for all other models can be found at 10.6084/m9.figshare.c.5006684. To validate our sampling method, we also obtained converging parameter estimates for model 1 using Stan with the RStan package (Stan Development Team 2018; Supplementary Figures S5 and S6). While the model fitted the data for most participants in both groups well, the fit to the children’s data (Figure S3) shows some underestimation of the range of accuracy and RT in some quantiles. The group mean posteriors for each of the diffusion model parameters are presented in Fig. 6. Figure 7 shows estimates for drift rate (for each condition), boundary separation and non-decision time for each participant, as a function of age. These figures together show that adults and older children tend to have higher drift rates, narrower boundary separation and reduced non-decision time, compared to younger children. Starting point values were close to 0.5 (i.e. the starting point was roughly equidistant between the two bounds; see Fig. 6), which was expected as the direction of stimuli was counterbalanced. We expected starting point estimates to vary slightly between individuals (reflecting biases towards upward or downward stimuli), but we had no reason to suspect that they would vary systematically with age. Therefore, we did not investigate age-related differences in this parameter.

### Age-related Differences in Model Parameters in Children

To quantify age-related differences, we added age as a covariate relating to different model parameters in a series of models for the children’s data (see Table 1). Note that we do not present these models for adults, as no age-related effects were expected in these participants. However, the corresponding model results for adults can be found at 10.6084/m9.figshare.c.5006684. In model 2, we covaried age with drift rate, boundary separation and non-decision time. There was a positive standardised regression coefficient (posterior *M* = 0.45, posterior SD = 0.05) for drift rate, and smaller, negative coefficients for each of boundary separation (posterior *M* = − 0.10, SD = 0.03) and non-decision time (posterior *M* = − 0.10, SD = 0.02). This model had a lower DIC value (13,854.3) than model 1 (13,862.0), showing that the addition of age was justified by the gain in model fit.

However, we then compared model 2 with other models which had age relating only to a subset of these parameters. We did not run any models without age covarying onto drift rate, as drift rate had the largest regression coefficient with age in model 2. DIC favoured model 2 (13,854.3) over a model which had age covarying only with drift rate (model 3, DIC = 13,863.8) and a model which had age covarying with drift rate and non-decision time (model 5, DIC = 13,861.9). Indeed, models 3 and 5 had similar DIC values to the model without covariates (model 1), suggesting that in these cases, the increase in model complexity associated with the addition of age was not justified by the gain in model fit. However, the model with the lowest DIC value was a model which had age covarying with drift rate and boundary separation (model 4, DIC = 13,851.1). Although model 4 was close to model 2 in terms of DIC (a difference of 3.2), model 4 had a lower DIC value and fewer predictors compared to model 2, suggesting that model 4 was the best model. Accordingly, our model comparisons suggested that age has effects on drift rate and boundary separation (but not non-decision time), with older children having higher drift rates (coefficient posterior *M* = 0.45, posterior SD = 0.05) and narrower boundary separations (coefficient posterior *M* = − 0.10, posterior SD = 0.03) than younger children.

### EEG Correlates of Model Parameters

To investigate links between model parameters and EEG measures, we used each participant’s reliable component slope values as covariates relating to drift rate in models 6 to 8, for adults and children. In model 6, we entered the slope values from component 1 as a covariate relating to drift rate. In both children and adults, slope values were positively related to drift rate values (children: coefficient posterior *M* = 0.43, SD = 0.05; adults: coefficient posterior *M* = 0.47, SD = 0.12). In model 7, we entered the slope values from component 2 as a covariate relating to drift rate. Here, there was a much smaller, positive relationship between slope values and drift rates (children: coefficient posterior *M* = 0.06, SD = 0.06; adults: coefficient posterior *M* = 0.05, SD = 0.13). For both children and adults, this model had a lower DIC value (children: DIC = 13,855.9; adults: − 3642.5) than model 6 (children: DIC = 13,863.1; adults: − 3635.6), suggesting that component 2 was the best EEG predictor in both groups. In model 8, we entered slope values from both components as covariates relating to drift rate. The DIC values for model 8 (children: DIC = 13,857.3; adults: DIC = − 3636.8) were higher than those for model 7, with model 7 (including component 2 only) being the model with the lowest DIC among the models with EEG predictors for adult participants (DIC = − 3642.5) and child participants (DIC = 13,855.9).

In both groups, the model with the lowest DIC value was model 7. However, the residual variance of the regression model on drift rate was actually *higher* for this model (children: *σ*^2^_res_ posterior *M* = 0.76; adults: *σ*^2^_res_ posterior *M* = 0.81) than for model 6 (children: *σ*^2^_res_ posterior *M* = 0.56; adults: *σ*^2^_res_ posterior *M* = 0.60) or model 8 (children: *σ*^2^_res_ posterior *M* = 0.56; adults: *σ*^2^_res_ posterior *M* = 0.60). We suggest that the relatively small differences between these models in DIC may not be fully reliable; as the computation of DIC is based on posterior samples, sampling variation can render small DIC differences unstable. Instead, model 6 (with EEG component 1 only) appears to provide the best account of the data for both groups. Model 8 (which includes both EEG components) does not lead to clear reductions in DIC, suggesting that the inclusion of both EEG components as predictors for drift rates does not convey a sufficient gain in predictive performance to offset the added model complexity.

It is notable, however, that model 6 did not perform better in terms of DIC than model 1 (without covariates) in either children or adults. Therefore, our model comparison results suggest that the increased model complexity associated with the addition of EEG covariates is not justified by the increases in model fit. For children, the best performing model still appears to be model 4 (with age effects on drift rate and boundary separation), suggesting that age is a better predictor than EEG alone. In adults, the best performing model appears to be model 1 (with no covariates), again suggesting that no benefit in predictive accuracy is conferred by adding EEG into the model. This lack of benefit in predictive accuracy could be due to noise in the EEG measures (Hawkins et al. 2017).

### Combining Age and EEG Correlates

**Fig. 8:**
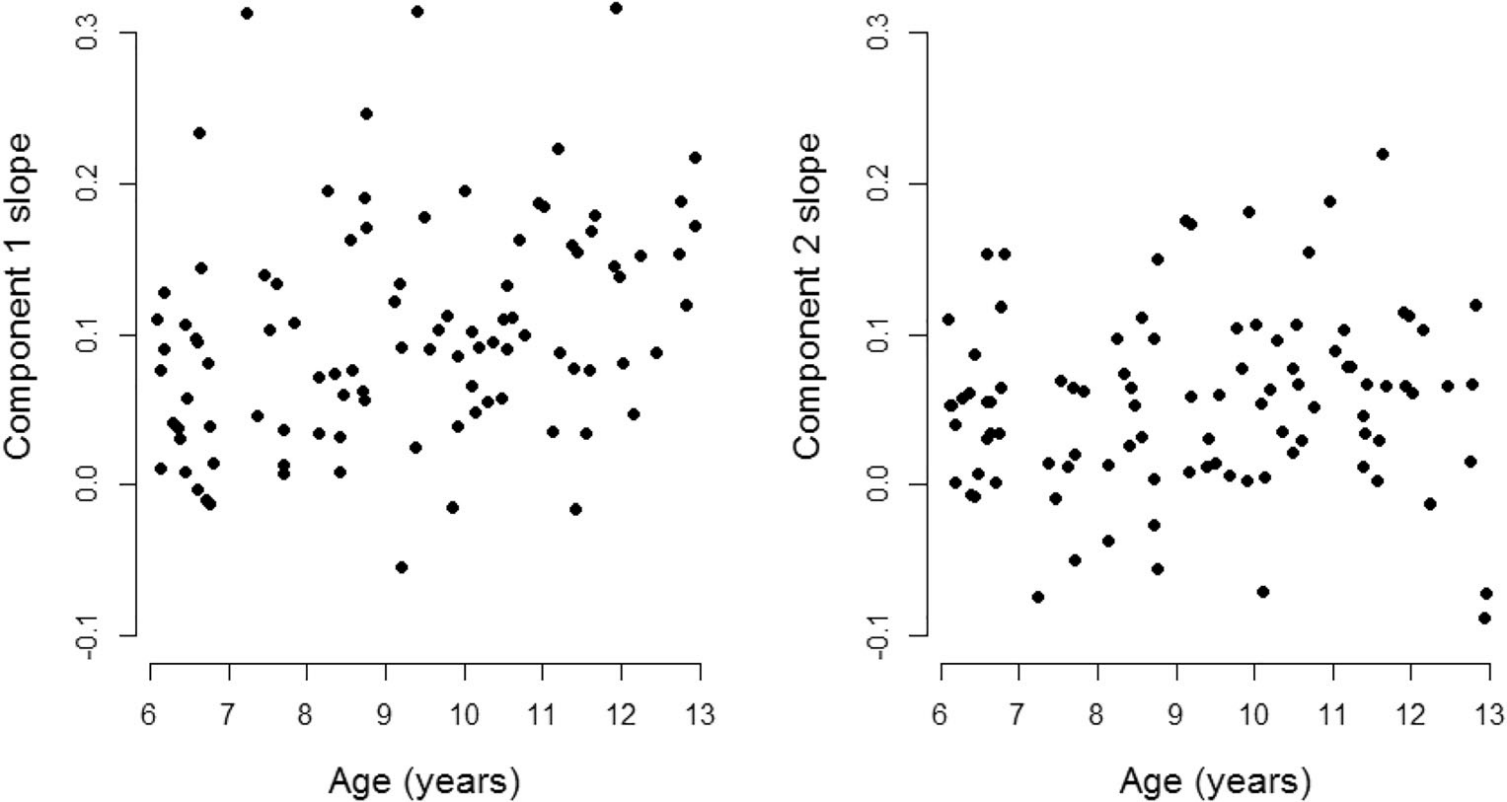
Age-related differences in slope values of EEG components 1 and 2 in the child group

**Fig. 9:**
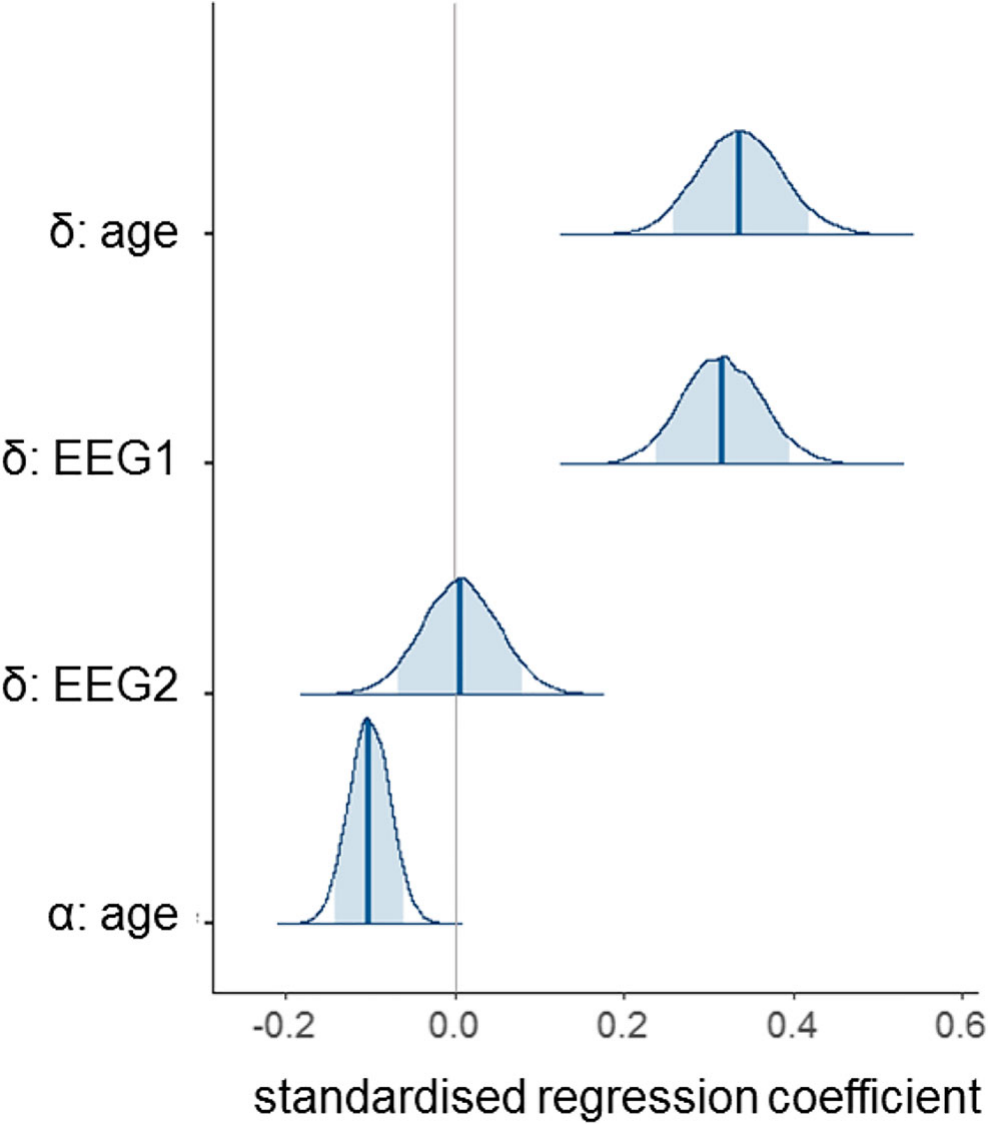
Posterior distributions of estimated standardised regression coefficients from model 10 fit to children’s data. Posterior distributions of the four standardised regression coefficients in model 10: age, EEG component 1 (EEG1) and EEG component 2 (EEG2) covarying with drift-rate (*δ*), and age covarying with boundary separation (*α*). The thick lines represent the median point estimate, and the shaded regions represent the 90% probability mass

Having characterised the effects of age and EEG covariates on model parameters separately, we next sought to determine whether adding them into a model together would lead to improvements in DIC compared to entering either alone. Component 1 itself showed a positive relationship with age, as shown in Fig. 8 (component 1: *r* = 0.30, 95% CI = [0.11, 0.48]), with older children generally having steeper slopes than young children. In component 2, there was a much weaker positive relationship (*r* = 0.09, 95% CI = [− 0.11, 0.29]). We ran four additional models for the children’s data which included both age and EEG covariates (models 9–12). In models 9 and 10, we added age covariates into our model with two EEG covariates (model 8). In model 9, age covaried with drift rate, as well as with both EEG slope measures (components 1 and 2). In model 10, we also allowed age to covary with boundary separation, along with the other covariates in model 9. In models 11 and 12, we took the best model with only age covariates (model 4) and added the EEG components individually. Thus in model 11, the component 1 slope measure and age covaried with drift rate, and age covaried with boundary separation. In model 12, we instead entered the component 2 slope measure. Posterior distributions of the regression coefficients for the most complex model, with four covariates (model 10), are presented in Fig. 9.

Of these four models, the lowest DIC value was obtained for model 12 (DIC = 13,857.1), which had component 2 and age covarying with drift rate, and age covarying with boundary separation. However, the best model for children in terms of DIC was still a model which had only age covariates (model 4). The residual variances for models 4 and 12 were almost equal (in both models, *σ*^2^_res_ posterior *M* = 0.54 for drift rate and *σ*^2^_res_ posterior *M* = 0.05 for boundary separation), suggesting that both models fit comparably well. Given the additional complexity in model 12 and the lower DIC values, it seems that model 4 is indeed the best model of the children’s data. Therefore, the benefit of adding age and EEG measures together is not worth the additional model complexity from the increased number of parameters. It is possible that the measurement variance in our EEG data means that information is not gained once age is taken into account.

## Discussion

In this study, we applied hierarchical Bayesian diffusion models to decompose motion coherence response time and accuracy data from children and adults into underlying psychological processes. We investigated relationships between these processes (as quantified by model parameters) and age and EEG measures. We looked at age effects in two ways: first, through a parameter estimation approach within a single model and then through a model comparison approach. Our parameter estimation approach suggested age-related differences in drift rate, boundary separation and non-decision time. These effects could be seen both when comparing the posterior distributions for adults and children and when considering the children’s parameter estimates as a function of age. Younger children had lower drift rates than older children and adults, meaning that they accumulated sensory evidence at a reduced rate. Younger children also had wider response boundaries than older children and adults, reflecting increased response caution, and they had longer non-decision times. These age-related differences echo the findings of other studies which have compared adults and children in quite different tasks (e.g. numerosity discrimination, lexical decision and linguistic comprehension) (Nordmeyer et al. 2016; Ratcliff et al. 2012; Schneider and Frank 2016), suggesting that age-related changes in diffusion model parameters generalise across tasks to some extent.

Our model comparison approach suggested that the best model of children’s data (in terms of DIC) was a model which had age effects only on drift rate and boundary separation, and not on non-decision time. The results of the model comparison approach therefore agree with our parameter estimation approach for two key parameters of the model (drift rate and boundary separation), showing that the results for these parameters are robust. Conversely, age effects on non-decision time are less clear. The DIC value for the model with age effects on all three parameters (drift rate, boundary separation and non-decision time) was only marginally higher (3.2) than the model with age effects only on two parameters (drift rate and boundary separation). However, by giving preference to the model with the fewest parameters and the lowest DIC value, it seems that age-related differences related to the decision-making process are most important for predicting children’s behavioural responses in this task, rather than age-related differences in processes considered to be outside of the decision-making process, such as sensory encoding and response generation.

Importantly, our diffusion modelling approach uncovers age-related differences in latent psychological processes that are not evident when just considering accuracy and response time data alone. Reduced drift rates and increased boundary separation have similar effects on response time (making responses slower) but have opposing effects on accuracy (with reduced drift rates leading to *reduced* accuracy and increased boundary separation leading to *increased* accuracy). These opposing processes are therefore undetectable when considering accuracy alone, and only become apparent when jointly modelling accuracy and response time. Our study therefore provides insights into the psychological processes involved in children’s perceptual decision-making and how these vary as a function of age.

Using a data-driven component decomposition technique, reliable components analysis, we identified two response-locked components that resembled a decision-making process, in both adults and children. These components had ramping activity preceding the response. The most reliable component was maximal over centro-parietal electrodes, resembling the centro-parietal positivity that has previously been linked to drift rate (O’Connell et al. 2012; Kelly and O’Connell 2013). The second most reliable component was instead maximal over occipital electrodes. The topographies of these components were similar to the stimulus-locked components identified using the same data by Manning et al. (2019). The slope of ramping activity in both components became steeper as a function of age. Importantly, age-related differences in these slope values cannot be explained solely by differences in skull conductivity with age (Hoekema et al. 2001; Wendel et al. 2010; see Scerif et al. 2006, for review). The grand average waveforms of the children took a different shape to those of the adults, with the children’s components having a more protracted increasing positivity than the adults, particularly in component 1. Instead, age-related differences in skull conductivity would lead to a scaling factor being applied to the amplitudes across ages (and thus slopes appearing steeper in the child participants).

In line with our intuitions that the slope of the ramping activity in the components was related to the decision-making process, we found that both EEG components covaried with drift rate, with steeper slopes being linked to higher drift rates. Our finding of two components being related to drift rate is in line with Dmochowski and Norcia’s (2015) conclusion that multiple drifting processes contribute to the decision-making process. However, component 1 had a much larger effect than component 2, in line with previous research in adults linking the centro-parietal positivity to the drift rate (O’Connell et al. 2012; Kelly and O’Connell 2013). In the adults, a model with just component 2 covarying with drift rate had a lower DIC value than a model in which component 1 covaried with drift rate. However, the DIC values for these two models were relatively similar, and the residual variance was lowest for the model with component 1 covarying with drift rate. Similarly, in children, the model with the lowest DIC value among the models with EEG covariates was a model in which component 2 only covaried with drift rates, but the residual variance was higher than a model with just component 1 covarying with drift rate. We suggest that small differences in DIC may have resulted from sampling variability and note that DIC can be biased towards more complex models in nested model comparison (Evans 2019). In terms of minimising residual variance and model complexity, we suggest that a model with just component 1 covarying with drift rate provides the best fitting model with EEG covariates, for both groups.

Yet, according to our model comparisons, this model did not perform as well as a model with only age effects for the children and did not perform as well as a model without covariates for the adults. Therefore, it appears that the inclusion of the EEG covariates added model complexity that was not justified by the increase in model fit—perhaps as a result of measurement variance in the EEG measures (see Hawkins et al. 2017). However, the correlation between parameters and neural data is nonetheless of interest. Indeed, in younger, less able participants, overt responses may be harder to compute, but EEG markers are still measurable. Moreover, when studying individuals with different developmental conditions, the EEG data may provide additional information that may improve model fit.

As well as investigating the effects of age and EEG separately, we also investigated whether the model could be improved by including both age and EEG measures as covariates. Our model comparisons suggested that the best-fitting model for the children’s data was still the model with just age effects on drift rate and boundary separation. Together, our results suggest that there is no incremental information in the EEG data (once age is taken into account)—perhaps due to measurement variance in our EEG data, which might offset the gain in information above and beyond age.

Our modelling results suggest that more focus should be given to decisional processes when investigating children’s responses to perceptual information. In a similar vein, Braddick et al. (2016) reported that the surface area of brain regions involved in accumulating sensory evidence towards a decision bound were important for explaining individual differences in children’s coherent motion thresholds. As we have established that boundary separation varies with age, it is important to control for speed-accuracy tradeoffs when quantifying children’s perceptual sensitivity. Diffusion models, as used here, provide a way of assessing whether sensitivity is immature in children once accounting for differences in decision-making style. Our results suggest that children’s perceptual sensitivity changes with age in this task, even when controlling for speed-accuracy tradeoffs. Such age-related changes in perceptual sensitivity could reflect changes in averaging ability (Manning et al. 2014) and visual attention, including the ability to suppress noise (see Nunez et al. 2015, 2017). An open question is whether young children differ from adults in the extent to which they can flexibly adjust their boundary separation settings—for example in response to instructions to emphasise either speed or accuracy (see Mulder et al. 2010).

We note that our 4-parameter diffusion model did not fit all children’s data perfectly, with the model underestimating the range of accuracy and RT in some quantiles. This observation suggests that the diffusion model may be a less adequate model of decision-making for children than it is for adults, perhaps because children have been shown to make more attentional lapses than adults (Manning et al. 2018). Future work could explicitly model the proportion of observations resulting from a contaminant process (e.g. Dmochowski et al. 2012; Nunez et al. 2019). Future work could also investigate whether children’s responses may be captured better by models which incorporate urgency signals or collapsing boundaries (Cisek et al. 2009; Chandrasekaran and Hawkins 2019; Ditterich 2006; Hawkins et al. 2015). While the diffusion model has been shown to explain human adult’s data better than the urgency-gating model, at least in tasks where the evidence is kept constant through a trial (Hawkins et al. 2015; Evans et al. 2017), we know of no work to have investigated this issue in child participants.

Our study of typical development provides an important benchmark for understanding performance in atypical development. By decomposing performance into distinct components, diffusion models have the potential to identify the reasons why children with various developmental conditions perform differently to typically developing children and to uncover differences in processing which are not apparent in the response time and accuracy data alone (White et al. 2010). Moreover, we have demonstrated that diffusion model parameters can be linked to neural correlates in typically developing children—an approach which could be extended to understand the neural underpinnings of atypical perceptual processing in those with developmental conditions.

## Electronic supplementary material

ESM 1 (DOCX 122 kb)
