# Supplementary material for: Perceptual Decision-Making in Children: Age-Related Differences and EEG Correlates
Source: Comput Brain Behav. 2020 Jun 19;4(1):53–69. doi: 10.1007/s42113-020-00087-7 (PMC7870772; doi:10.1007/s42113-020-00087-7)
Supplement: Supplementary file 1 — (DOCX 122 kb) [file 42113_2020_87_MOESM1_ESM.docx]

**Supplementary Files**

**
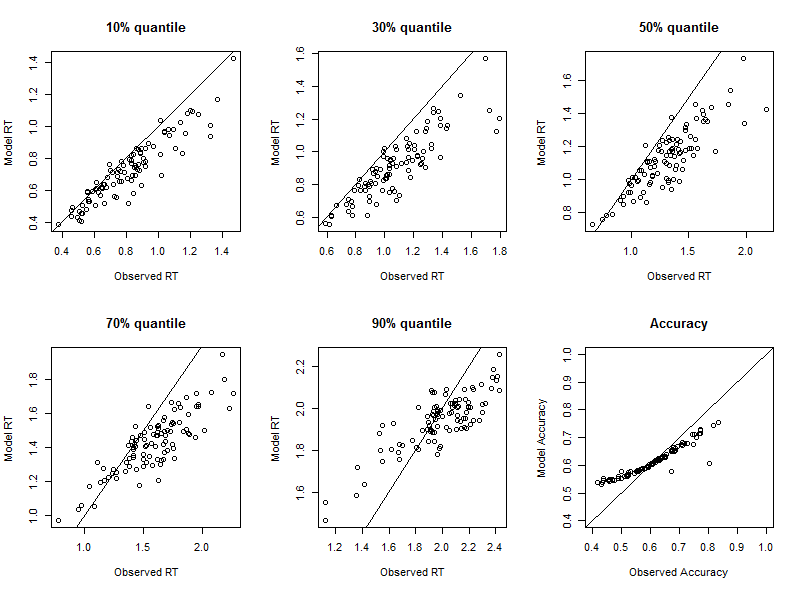
**

**Figure S1: Posterior predictive plots for model fit to children’s data in condition 1 only**

Observed response times for each quantile (10%, 30%, 50%, 70%, 90%) for correct trials and observed accuracy for each participant plotted against those predicted by the model, for the 10% coherence condition (which we excluded from the main analysis). Prior to fitting the model, we conducted Exponential Weighted Moving Average filtering on all 4 conditions, and removed 3 child participants who had < 60% of data retained, leaving 97 children retained in the dataset. We ran a basic diffusion model on the filtered data from the 10% coherence condition, only.

**
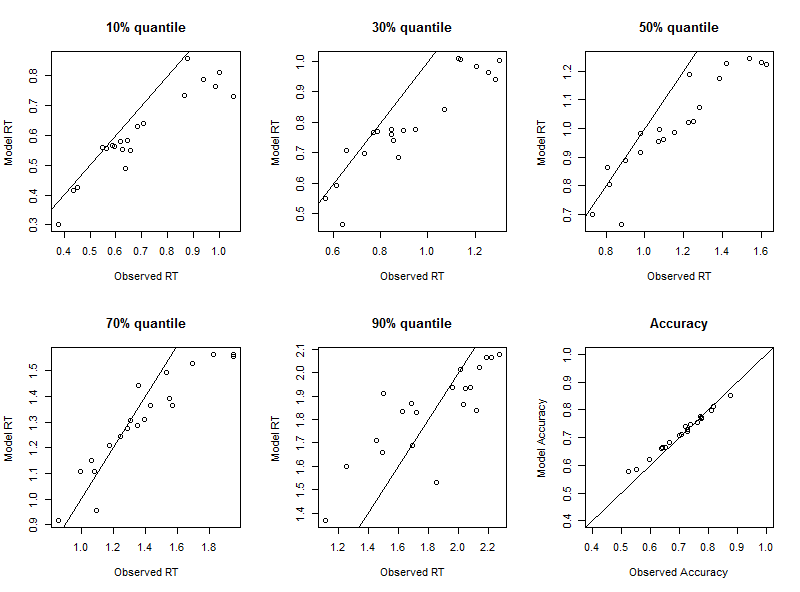
**

**Figure S2: Posterior predictive plots for model fit to adult’s data in condition 1 only**

Observed response times for each quantile (10%, 30%, 50%, 70%, 90%) for correct trials and observed accuracy for each participant plotted against those predicted by the model, for the 10% coherence condition (which we excluded from the main analysis). Prior to fitting the model, we conducted Exponential Weighted Moving Average filtering on all 4 conditions. We ran a basic diffusion model on the filtered data from the 10% coherence condition, only.

**
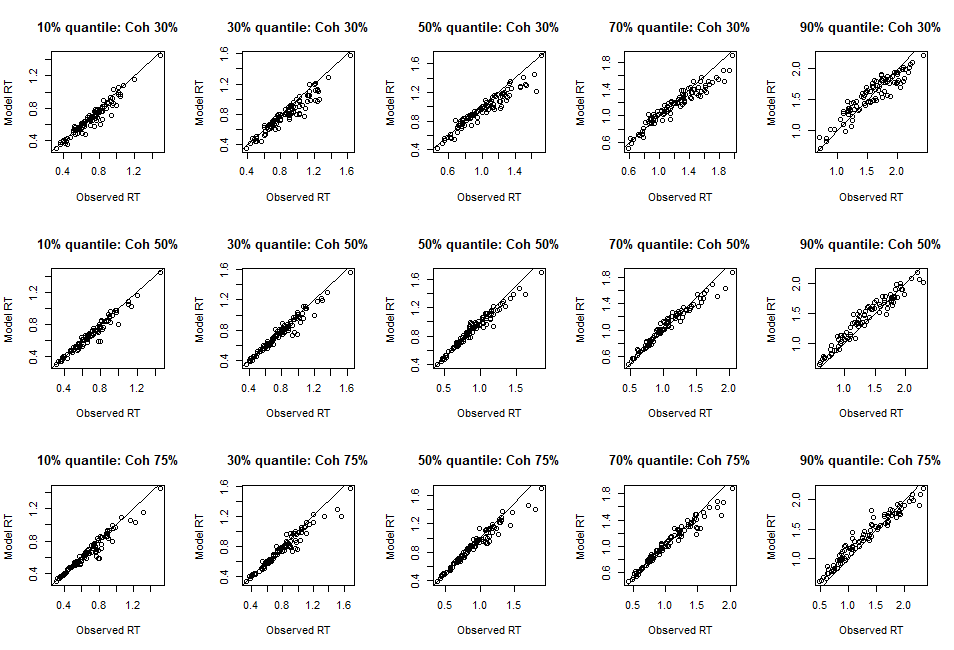

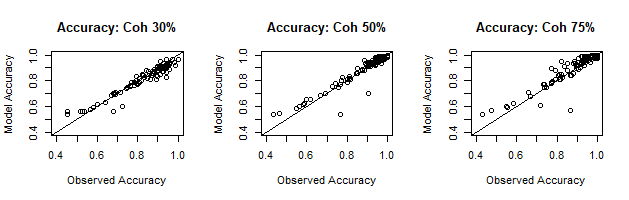
**

**Figure S3. Posterior predictive plots for Model 1 fit to children’s data**

Observed response times for each quantile (10%, 30%, 50%, 70%, 90%) for correct trials and observed accuracy for each participant plotted against those predicted by Model 1, for each coherence condition (‘Coh’: 30%, 50%, 75%).

**
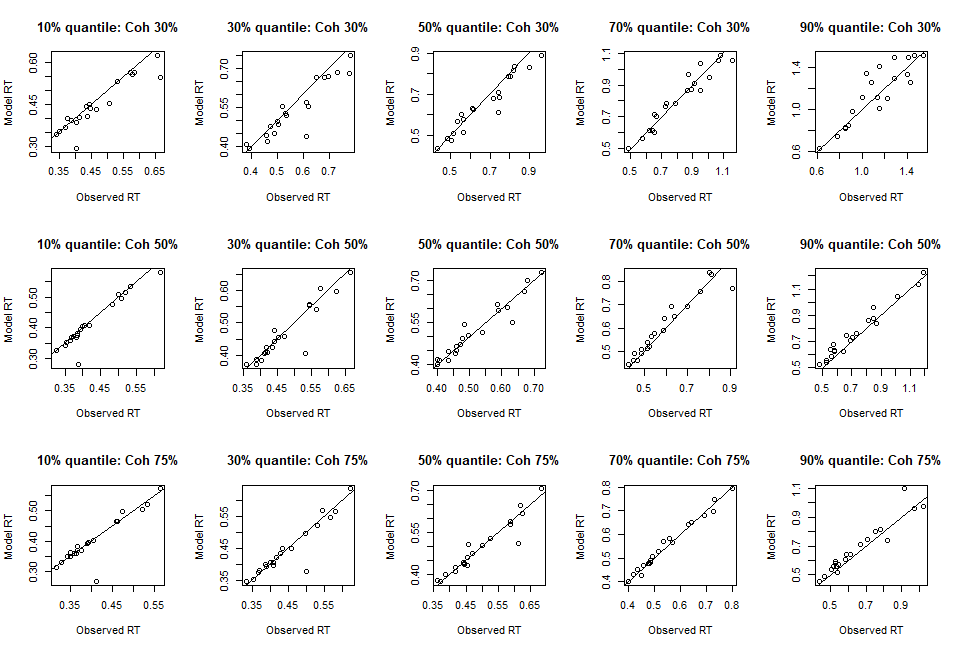

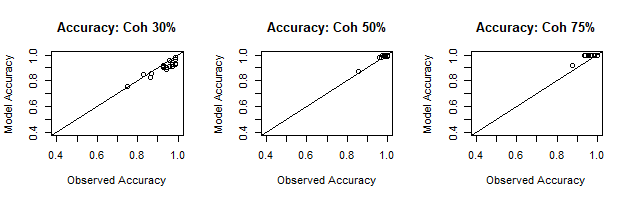
**

**Figure S4. Posterior predictive plots for Model 1 fit to adult’s data**

Observed response times for each quantile (10%, 30%, 50%, 70%, 90%) for correct trials and observed accuracy for each participant plotted against those predicted by Model l, for each coherence condition (‘Coh’: 30%, 50%, 75%).

**
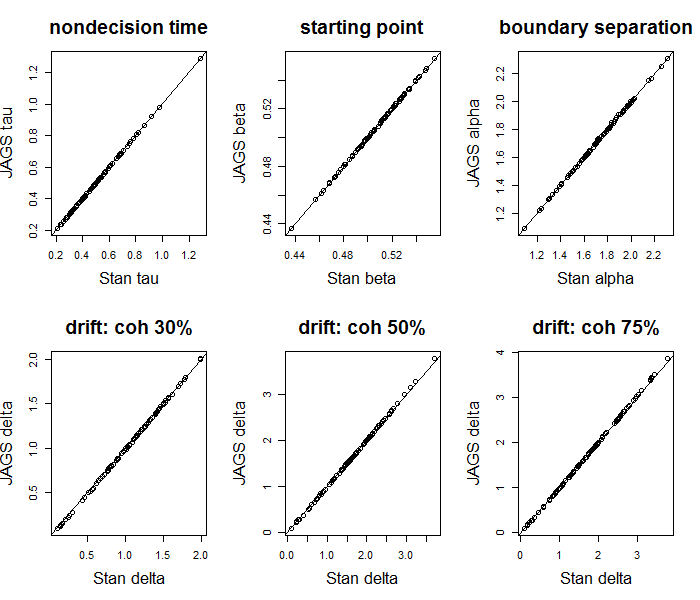
**

**Figure S5. Comparison of posterior mean parameter estimates for each child obtained from Model 1 fit using JAGS (as reported in the paper) and Stan**

Stan estimates were obtained using 3 chains each with 10,000 iterations and 4000 burn-in samples and thinning of 5.


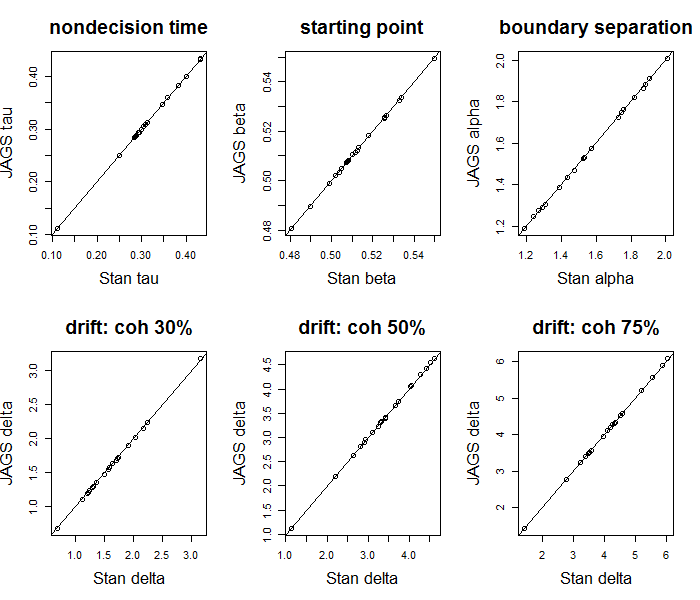


**Figure S6. Comparison of posterior mean parameter estimates for each adult obtained from Model 1 fit using JAGS (reported in the paper) and Stan**

Stan estimates were obtained using 3 chains each with 10,000 iterations and 4000 burn-in samples and thinning of 5.
